# Refined circular autopsy saw design and standardized technique for postmortem human temporal bone procurement and perilymph collection

**DOI:** 10.3389/fnins.2026.1893053

**Published:** 2026-09-18

**Authors:** Sophie S. Jang, Sandra E. Torres, Shelley Batts, Konstantina M. Stankovic

**Affiliations:** 1 Department of Otolaryngology – Head and Neck Surgery, Stanford University School of Medicine, Stanford, CA, United States; 2 Department of Neurosurgery, Stanford University School of Medicine, Stanford, CA, United States; 3 Wu Tsai Neurosciences Institute, Stanford University, Stanford, CA, United States

**Keywords:** body mechanics, circular saw, human inner ear, otopathology, perilymph, postmortem, temporal bone autopsy saw, temporal bone plug

## Abstract

Postmortem human temporal bones provide an irreplaceable resource for studying the cochlea and vestibular labyrinth in their native anatomic context, as the tissues of these organs cannot be non-destructively biopsied. However, temporal bone procurement using an intracranial circular saw blade remains technically challenging, with intermittent blade fractures despite iterative optimization of blade materials and design. Because specimens are rare, efficient collection of biologic materials, including perilymph, further increases the value of each autopsy. We qualitatively assessed modifiable and non-modifiable factors contributing to blade fracture risk during five postmortem temporal bone collections by three operators and evaluated refinements for perilymph collection. Potential contributors, based on direct observation and operator-reported experience, included irregular middle cranial fossa (MCF) topography, angle of saw blade engagement with bone, cortical scoring technique, and operator body mechanics. Modifiable factors included maintaining an orthogonal saw axis relative to the MCF, performing cortical scoring before complete osteotomy, and repositioning of the operator to improve ergonomic alignment with the saw axis. Non-modifiable factors included the irregular surface of the MCF, temporal bone asymmetry, and physical constraints limiting orthogonal saw alignment. These observations informed a refined saw blade design intended to accommodate non-modifiable anatomic and ergonomic constraints. Perilymph workflow refinements enabled timely recovery of low-volume samples before temporal bone fixation. After optimization, trainee procurement duration averaged 8.0 ± 2.5 min across five serial sessions, whereas experienced operators retrospectively self-reported ~5 min per temporal bone. These refinements are intended to improve the robustness, reproducibility, speed, and accessibility of postmortem temporal bone collection for human otopathology studies.

## 1 Introduction

Hearing and balance disorders are highly prevalent sensory conditions that substantially impair communication, mobility, and quality of life (Agrawal et al., 2018; World Health Organization, 2026; Hülse et al., 2019). Their effects are often permanent because the human cochlea and vestibular system have limited regenerative capacity after injury (Hudspeth, 2014; Atkinson et al., 2015). Yet human inner ear pathology remains difficult to study directly because the cochlea and vestibular labyrinth are fragile, deeply embedded within the temporal bone, and contain sensory cells below the resolution of clinical imaging modalities (Rask-Andersen et al., 2012; Batts et al., 2025). Human temporal bone otopathology therefore remains one of the few methods for linking hearing and balance disorders to cellular and anatomic changes within the human inner ear (Sagi et al., 2023; da Costa et al., 2018).

Despite its value, temporal bone procurement is limited by autopsy availability, variable postmortem interval (PMI), and the specialized tools and expertise required for specimen harvest and processing (Paratz et al., 2023; Harrington and Sayre, 2010). These constraints underscore the need to refine investigator-controlled steps, including harvest technique, instrumentation, specimen handling, and collection of biologic materials such as perilymph (Sagi et al., 2023). Nadol et al. described temporal bone collection methods and the temporal bone plug technique in the 1990s (Nadol, 1996), but the Stryker autopsy saw and plug cutter used for this approach are no longer commercially available (Sagi et al., 2023; Nadol, 1997; Schuknecht, 1968). Investigators have since fabricated circular saw blades based on Nadol’s design, and Sagi et al. (2023) described an alternative blade and further refined the plug method. However, intermittent blade fracture remains a procedural risk that delays harvest, wastes materials and operator time, and potentially damages rare human specimens. We therefore sought to refine the current procurement procedure and circular saw blade design.

Persistent fractures suggest that blade durability depends not only on material and design, but also on skull base anatomy and operator technique. The middle cranial fossa (MCF), the primary cutting surface during temporal bone plug procurement, is irregular and anatomically variable, complicating perpendicular saw alignment during the initial stage of the osteotomy. Operator-dependent factors, including height relative to the evisceration table, handedness, stance, and force alignment, may further contribute to off-axis loading. Although prior articles, protocols, and videos describe temporal bone removal and anatomic saw placement (Sagi et al., 2023; Nadol, 1996; Mass Eye and Ear, n.d.), guidance on body mechanics and force alignment during plug extraction remains limited.

Temporal bone procurement also provides an opportunity to collect perilymph, a microliter-scale inner ear fluid increasingly used for biomarker discovery and molecular characterization of otologic disease (Lysaght et al., 2011; Lin et al., 2019; Schmitt et al., 2021; Warnecke et al., 2019). Because the typical volume of human perilymph sampled through the round window is small [1–10 μL (Schmitt et al., 2017)] and its composition may be affected by sampling location, dilution, or contamination, efficient collection methods are needed to preserve sample quality and maximize the scientific value of each autopsy (Salt et al., 2006).

Here, we describe refinements to postmortem temporal bone procurement and perilymph collection intended to improve robustness, reproducibility, speed, and accessibility of the workflow. We qualitatively assessed modifiable and non-modifiable contributors to circular saw blade fracture, refined the blade design to better accommodate anatomic variability and reduce off-axis force, and described postmortem-specific workflow refinements for cochlear and vestibular perilymph collection.

## 2 Materials and methods

### 2.1 Postmortem human specimens and ethical approval

Human temporal bones were collected postmortem through the Research Autopsy Center at Stanford University, which obtained informed consent for research use from all donors prior to death and donors’ next of kin postmortem. The study was determined to be exempt from Institutional Review Board review because research involving deceased individuals does not meet the federal definition of human subjects research. All specimens and data were deidentified and the study was conducted in accordance with applicable institutional policies and ethical principles.

#### 2.1.1 Cohorts

Three evidence sources were used. Cohort 1 included five donors yielding 10 bilaterally collected temporal bone plugs across five autopsy sessions. All plugs were included in qualitative osteotomy assessments over the 5 directly observed serial procurement sessions. Procurement duration was measured by autopsy session/donor as the interval between completion of the first and second osteotomies during bilateral procurement. Four plugs from two rapid-autopsy donors (PMI < 12 h) were used for bilateral cochlear perilymph collection. A representative workflow timeline is provided in Supplementary Methods.

Cohort 2 comprised retrospective interviews with three operators regarding 16 prior bilateral procurement cases (32 temporal bones). These accounts provided contextual information on historical workflow and blade fractures and were not treated as directly observed or measured outcomes. Cohort 3 comprised two fixed temporal bones used for preliminary testing of the refined blade.

### 2.2 Evaluation of the temporal bone plug osteotomy

#### 2.2.1 Variability in temporal bone plug anatomy

Temporal bone plug osteotomies were planned using the internal auditory canal (IAC) and arcuate eminence (AE) as landmarks for circular saw placement, with the goal of preserving the cochlea and vestibular labyrinth within the specimen (Figure 1A). The circular saw blade was positioned perpendicular to the MCF floor; the superior surface of the resulting plug included the MCF floor and posterior petrous temporal bone in the posterior cranial fossa (PCF).

**Figure 1:**
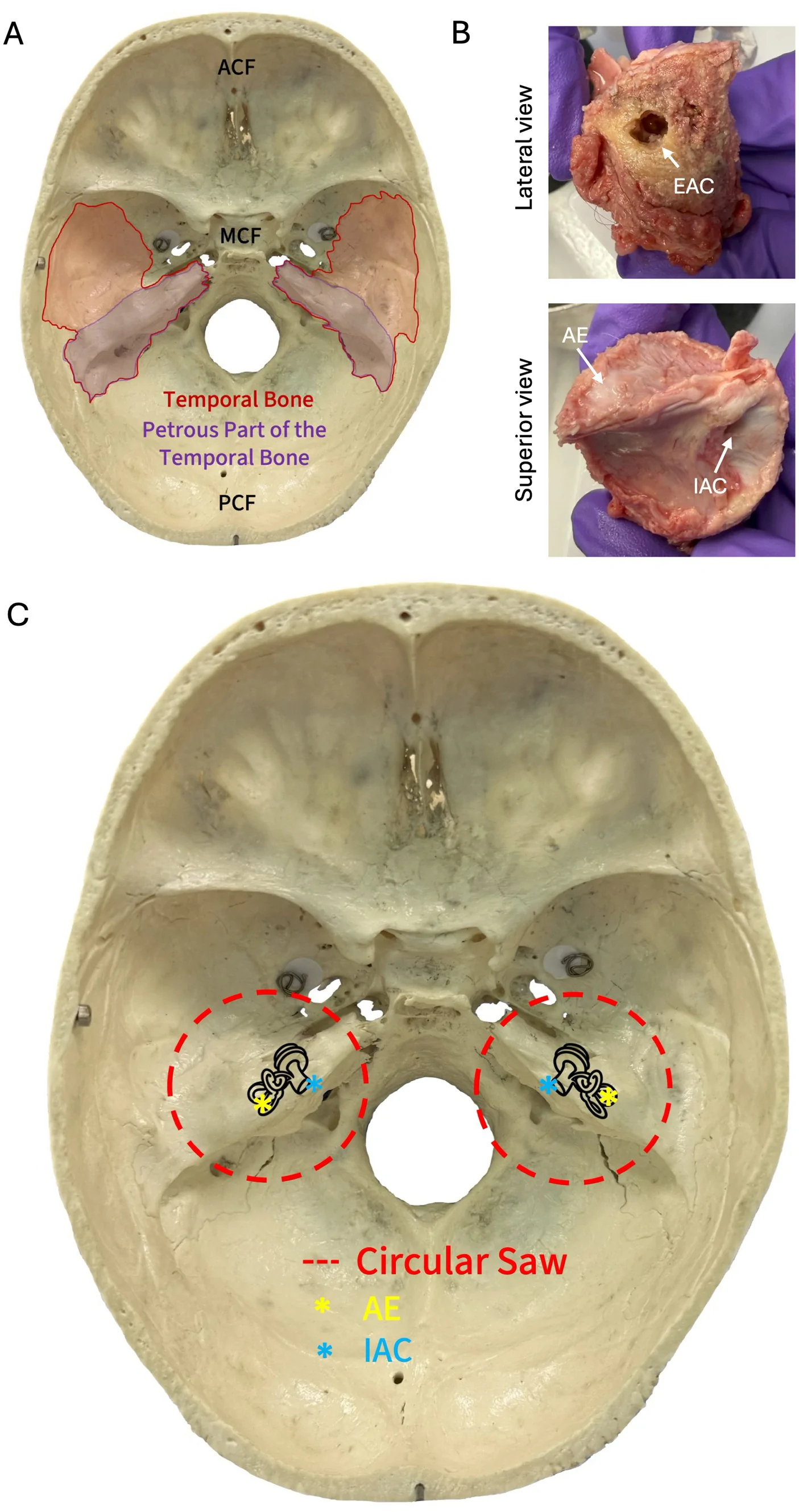
The Internal auditory canal (IAC) and the Arcuate eminence (AE) are the two main anatomical landmarks used for placing the circular saw. **(A)** The inner ear is embedded in the petrous part (purple) of the temporal bone (red) within the Middle cranial fossa (MCF). **(B)** Landmarks on the temporal bone plug include the External auditory canal (EAC) laterally, the IAC on the PCF wall, and AE on the MCF floor medially. **(C)** The circular saw is positioned to include the IAC (blue dots) medially and AE (yellow dots) laterally, as shown by the red circle. ACF, anterior cranial fossa; PCF, posterior cranial fossa.

We qualitatively assessed sources of variability in temporal bone plug procurement, distinguishing modifiable factors, defined as operator- or workflow-dependent features that could be adjusted, from non-modifiable factors, defined as inherent anatomic or procedural constraints (Supplementary Material). To contextualize expected anatomic heterogeneity, we reviewed the literature on intracranial topography and inner ear orientation. Left and right temporal bone plugs from five donors were visually compared to assess plug-shape consistency across individuals and bilateral symmetry within donors. The percentage of MCF captured in each plug was evaluated descriptively using ImageJ, using repeated linear measurements by one investigator.

To identify contributors to intermittent circular saw blade fracture, 10 serial osteotomies performed by three operators with similar expertise were qualitatively observed visually, in-person, from behind the operator and from the lateral view. Factors considered included the angle of the saw-axis relative to the MCF cutting plane, operator height and handedness, recurring fracture patterns, and cortical scoring technique during initial decortication. Operators were not blinded to the purpose of observation.

#### 2.2.2 Temporal bone procurement duration

To evaluate whether procedural refinement shortened procurement duration, temporal bone plug harvest time was quantified during five serial procurement sessions performed by one trainee operator after completing training and commencing autonomous procurement. Procurement duration was estimated as the interval between completion of the first and second temporal bone osteotomies within the same autopsy session. These data were used descriptively to evaluate change in procurement duration with operator experience; formal statistical testing was not performed because timing data were available for only one operator across a small number of sessions. Historical procurement duration before workflow optimization was estimated from operator experience and prior laboratory practice.

### 2.3 Circular saw blade refinement and fabrication

To address anatomic and operator-related contributors to blade fracture, the previously described design (Sagi et al., 2023) was refined. Prior optimized blades were produced by manual machining in an institutional machine shop (Stanford Machine Shop, CA, USA), making replacement blade production dependent on machinist availability and expertise, potentially introducing variability across blades. The original Stryker blade was also re-evaluated to identify design parameters that could be adapted using currently available manufacturing approaches. The effect of the refined blade design on fracture risk was inferred from observed contributors to off-axis loading and staged blade engagement.

With the goal of improving reproducibility, scalability, and accessibility, we developed a standardized manufacturing workflow suitable for either in-lab fabrication or turnkey production by an external machining vendor. The refined blade was designed in Fusion (Autodesk, v2702.1.58 arm64, CA, USA) using computer-aided design (CAD) for dimensional control and computer-aided manufacturing (CAM) to define machining specifications (CAD file available at Zenodo DOI: 10.5281/zenodo.20384380). Potential designs were evaluated *in silico* for volumetric compatibility using 3D Slicer (Surgical Planning Laboratory, v5.6.1, MA, USA) and a standardized digital cranial phantom. The final blade was fabricated by computer numerical control (CNC) machining. Because CAM parameters are CNC system-specific, machining specifications should be generated independently for fabrication.

### 2.4 Perilymph collection during temporal bone procurement

#### 2.4.1 Considerations for perilymph collection workflow

For standard otopathology, temporal bone plugs are typically placed immediately into fixative (e.g., 4% paraformaldehyde) to minimize the PMI, defined as the interval from donor death to fixation or experimental use. When perilymph collection is planned, temporal bone specimens are not fixed or immersed in solution before sampling to avoid perilymph dilution and contamination through the cochlear aqueduct or round window membrane. Instead, specimens are placed on ice immediately after procurement.

Because perilymph is a very low-volume fluid and collection must be completed before temporal bone fixation, we evaluated the workflow to identify modifiable steps that could reduce sampling time, preserve sample quality, and maximize sample recovery. Qualitative assessment focused on the preparatory sequence, communication between the procurement and preparation teams, equipment readiness and redundancy, specimen orientation, access to the perilymphatic space, and transfer of aspirated perilymph from the needle to the collection tube. These methods are intended as workflow descriptions rather than as evaluated refinements.

#### 2.4.2 Cochlear and vestibular perilymph sampling

The sampling approach was selected according to the target perilymph compartment, further described in Section 3.5. Here, we bilaterally collected cochlear perilymph through a transcanal, round window approach from a subset of Cohort 1 (*n* = 2 donors, 4 total samples). A MCF, superior semicircular canal (SCC) approach, used for vestibular perilymph collection, was also performed in each case up to the needle insertion step to assess the surgical procedure. Irrigation, when needed, was performed with saline. Further details are in Supplementary Methods.

## 3 Results

### 3.1 Anatomic variability of the MCF affects temporal bone plug osteotomy

We evaluated modifiable and non-modifiable contributors to variability in temporal bone plug procurement, focusing on three interacting components: the skull base cutting surface, the circular autopsy saw, and the operator. The literature review showed that prior studies conducting topographical mapping of the MCF reported it to be more irregular and complex than the anterior or posterior cranial fossae, with numerous neurovascular and nerve grooves, foramina, and bony prominences (Peris-Celda et al., 2019; Liu and Yi, 2020; Teresi et al., 1987; Rosas et al., 2014; Dayoub et al., 2010; Salma et al., 2013). Based on anatomic review, visual assessment of procured plugs, and direct observation during osteotomy, the MCF topography was identified as a key non-modifiable contributor because of its irregular, non-planar surface and variable relationships to the inner ear (Peris-Celda et al., 2019; Teresi et al., 1987; Figure 1). This topography prevents uniform perpendicular engagement of the circular saw blade during initial cortical scoring and can produce intermittent non-orthogonal contact between the blade and bone (i.e., acute or obtuse angle between the MCF and saw axis). Direct intraoperative observations and operator-reported experience suggested that an incorrect cortical scoring angle may establish a non-orthogonal trajectory that persists throughout the osteotomy.

In Cohort 1, temporal bone plug shape varied across donors and between left and right sides within donors, despite using consistent landmarks for saw placement. The proportion of MCF captured averaged 55.7% ± 8.5% and ranged from 36.7 to 64.6% across 10 plugs procured by three operators. All temporal bone specimens were visually inspected to confirm inclusion of key landmarks, including the IAC and AE (Figures 1B,C), to be intact, and that no breach of the cochlea or vestibular labyrinth occurred.

### 3.2 Operator-dependent factors hypothesized to contribute to circular saw blade fracture risk

Ten osteotomies from five bilateral donor procurements across three operators were directly observed from the lateral view and from behind the operator to identify modifiable contributors to intermittent blade fracture. From the lateral view, the saw axis was non-orthogonal to the MCF cutting plane in 4 of 5 observed cases (Figure 2).

**Figure 2:**
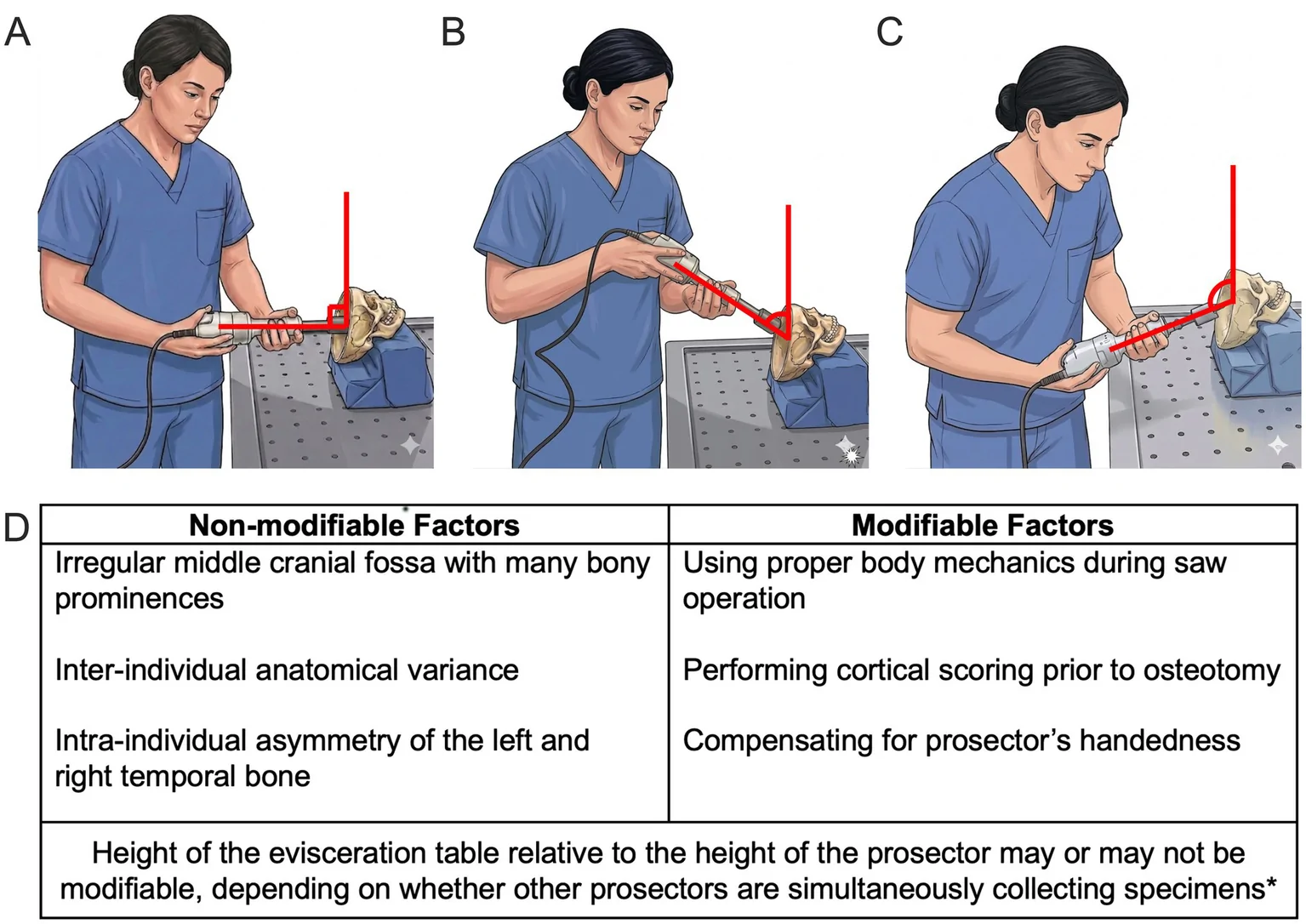
Proper body mechanics when performing the osteotomy may decrease the risk of saw fracture and ensure the inner ear is contained in the procured temporal bone plug. **(A)** The axis of the saw should be perpendicular (90°) to the axis of the floor of the middle fossa. **(B)** If the angle between the axis of the saw and the middle fossa is too small (<90°), there is a risk of cutting through the cochlea anteriorly. **(C)** If the angle between the axis of the saw and the middle fossa is too large (>90°), procurement time may be prolonged due the anterior deviation of the axis traversing through a larger amount of bone requiring additional osseous reduction. **(D)** Non-modifiable and modifiable factors identified as contributory to the circular saw blade fracture. * Denotes that if the height of the table is non-modifiable, a prosector of shorter stature may use a stepping stool while a tall prosector may use an adjustable chair or a wider stance. Panels **(A–C)** generated using Gemini 3 Flash Image.

Operator height relative to the evisceration table appeared to influence saw angle: taller operators tended to use a smaller angle relative to the MCF (Figure 2B), whereas operators of shorter stature tended to use a larger angle (Figure 2C). Because table height may not always be modifiable, particularly when multiple prosectors are working simultaneously, operator stance and positioning were identified as modifiable compensatory factors.

Across 10 osteotomies performed during the observed procurement sessions using the non-optimized blade, one minor circular saw blade fracture (<25% circumference of the saw blade) occurred when a left-handed operator attempted to adjust the saw-axis trajectory mid-osteotomy while harvesting the left temporal bone. Visual inspection of the specimen showed no violation of the inner ear organs. To contextualize this event, we obtained retrospective accounts from two experienced operators regarding prior blade fractures outside the observed sessions and identified two additional fractures during right temporal bone harvest by a right-handed operator. Together, these events suggested that operator handedness and laterality may contribute to off-axis loading, particularly when the dominant arm adducts inward during osteotomy. An osteotomy demonstration using proper body mechanics for one- and two-person procedures is shown in Supplementary Video 1.

Direct observation suggested that cortical scoring was an important procedural step in performing the osteotomy orthogonally to the MCF. This technique, which creates a guide groove directing subsequent blade advancement, varied across operators and appeared to influence osteotomy control. Observed approaches included activating the saw before bone contact and using small pulsing movements to create a guide groove, activating the saw only after the blade contacted bone, and omitting cortical scoring. When the oscillating autopsy saw has a static start against the cutting surface, the high impact loading and uncontrolled kickback of the saw can lead to sub-optimal saw advancement trajectory, which damages both the saw blade and the bone. When cortical scoring was not performed, we observed greater potential for blade stress due to cutting at the incorrect axis or incorrect margin containment and then attempting axis adjustment mid-osteotomy. These findings support standardized cortical scoring before initiation of the osteotomy to improve margin definition, maintain orthogonal alignment, and reduce blade stress. All modifiable and non-modifiable factors identified are summarized in Figure 2D.

### 3.3 Refined circular saw blade design accommodates non-modifiable anatomic constraints

Several prior saw blade iterations aimed to increase fracture resistance by modifying the blade material and saw tooth dimensions (Sagi et al., 2023). To further address hypothesized contributors to blade stress, we refined its design to better accommodate non-modifiable anatomic features of the MCF. Because the circular saw cannot remain uniformly perpendicular to the irregular MCF surface throughout the cut, applied force is decomposed across two axes rather than one perpendicular axis, creating shear stress that may increase fracture risk (Figure 3A).

**Figure 3:**
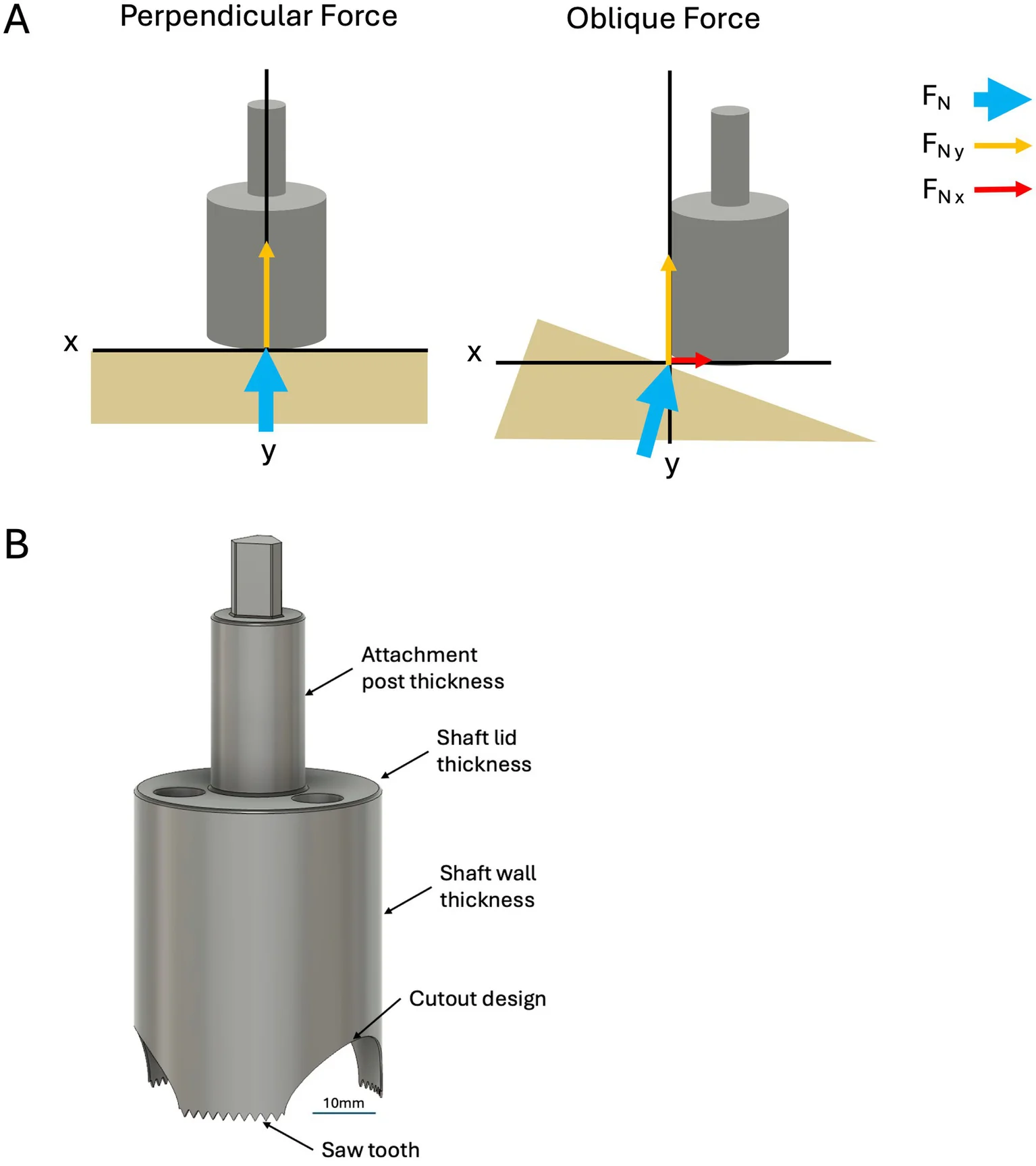
Conceptual model of a modified circular saw blade design to address sources of blade stress from non-orthogonally applied force. **(A)** The circular autopsy saw should ideally be used perpendicularly to the cutting surface, which results in a normal force (*F*_N_) along one coordinate axis. However, when the saw is not perpendicular to the cutting surface, the *F*_N_ is decomposed into the orthogonal components (*F*_Ny_, *F*_Nx_), leading to shear stress on the saw and increasing the likelihood of fracture. **(B)** The modified saw design included purposeful gaps in the blade to accommodate the uneven bony surface of the middle cranial fossa.

We therefore evaluated blade features that could increase maximal circumference engagement of the saw blade with cortical bone. Because the entirety of the circular blade cannot lie flat on the MCF due to the bony prominences, a segmented circular profile was designed (Figure 3B). Incorporating cutouts along the cutting edge was expected to permit staged engagement during cortical scoring, allowing localized contact followed by incremental enlargement of the guide groove until a uniform cutting depth was achieved. Further, the cutouts allowed for an escape space for accumulated bone debris and were intended to reduce blade loading. The CAD file for the refined blade is available in Supplementary File 1.

The blade design was additionally modified by changing the material to 420 stainless steel, reducing its thickness, increasing the number of saw teeth with decreased thickness to emulate the original Stryker saw blade, and adding post-machining polishing and passivation (machined by Fictiv, CA, USA). The post-machining treatment of the saw blade is meant to prolong integrity with repeated use with sterilization using an enzymatic detergent (Steris Prolystica Enzymatic Presoak and Cleaner, OH, USA) and scrubbing with concentrated bleach. The saw blade remains compatible with the oscillating autopsy saw (Stryker, model 810, MI, USA).

Preliminary testing of the refined blade in Cohort 3 suggested that osteotomies could be completed without blade fracture, difficulty with cortical scoring, excessive heat generation, or clogging. We noted that there may be minor differences in the characteristics of tissue specimens fixed prior to osteotomies and further testing in non-fixed temporal bones is needed.

### 3.4 Procurement duration decreased with greater trainee experience and optimized workflow

Procurement duration was evaluated using two evidence sources. First, experienced operators’ accounts of osteotomies performed in Cohort 2 indicated that, after optimization, procurement duration was approximately 5 min per temporal bone. Second, procurement-duration data were measured for one trainee operator across five serial, unilateral procurement sessions (Cohort 1) after training. In this trainee, the interval between completion of the first and second temporal bone osteotomies decreased from 11 min in the first session to 5 min in the fifth session, corresponding to a 54.5% reduction. Across the five sessions, procurement duration averaged 8.0 ± 2.5 min, with chronological session times of 11, 8, 10, 6, and 5 min.

### 3.5 Perilymph collection workflow refinements

#### 3.5.1 Refinements to maximize sampling efficiency

Perilymph collection, performed in a subset of Cohort 1, was evaluated as an additional opportunity to maximize the scientific value of each temporal bone procurement, while minimizing the duration of this added procedure. The main modifiable sources of delay were related to preparation and coordination rather than aspiration itself, including communication about the transport of the temporal bone plug to the perilymph collection station and prolonged duration retrieving replacement equipment. Postmortem sampling also allows surgical approaches to be adapted without the constraints of preserving physiological function or hemostasis. Mounting the temporal bone plug in a dissection bowl enables multi-axial rotation, improving visualization and access of target landmarks without extensive osseous reduction of the posterior external auditory canal wall. Based on operator experience after workflow refinement, perilymph sampling could typically be completed within approximately 7–10 min per ear once the specimen was mounted. This estimate was treated as an operator-reported workflow benchmark rather than a prospectively timed outcome. Fixation of the specimen is not recommended during preliminary training sessions of new operators, as it noticeably changes visual and tactile properties that negatively affect landmark identification.

#### 3.5.2 Cochlear and vestibular perilymph sampling approaches

The sampling approach was selected according to the target perilymph compartment. Cochlear perilymph was accessed through a transcanal, round window approach into the scala tympani (Figure 4A), whereas vestibular perilymph was accessed from the MCF, SCC approach (Figure 4B). Full surgical procedures for both approaches are provided in Supplementary Methods, and cochlear perilymph sampling is demonstrated in Supplementary Video 2.

**Figure 4:**
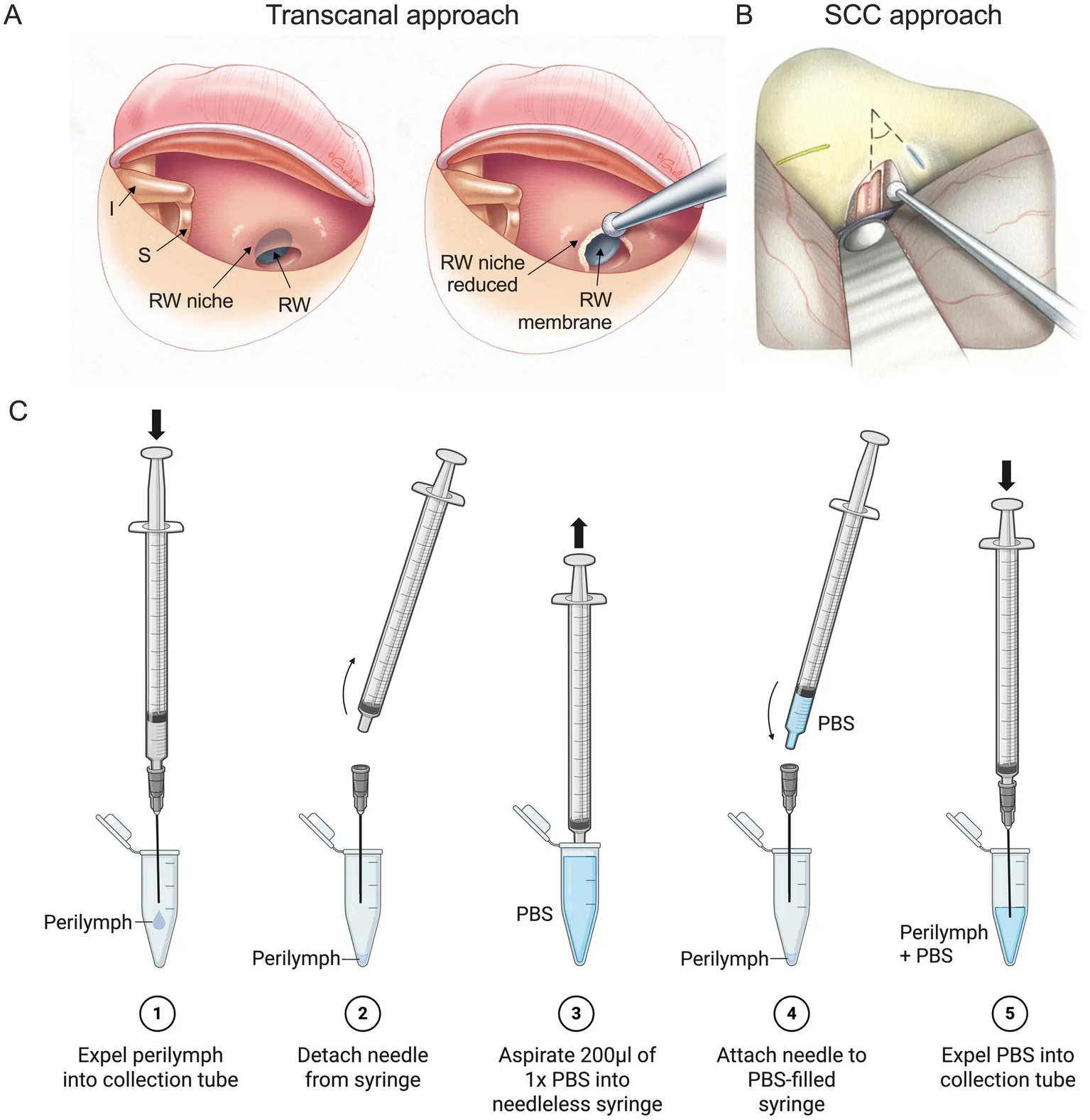
Cochlear perilymph can be collected via the transcanal, round window approach and vestibular perilymph can be collected via the middle cranial fossa, superior semicircular canal (SCC) approach. **(A)** The transcanal approach is performed through the external auditory canal on the lateral surface of the temporal bone plug. The tympanomeatal flap is elevated (left subpanel), the surgical field is widened if needed, the round window niche is drilled away (right subpanel), and an aspiration needle is inserted through the round window (RW) membrane. **(B)** The SCC approach is performed on the floor of the middle cranial fossa. The projected region of the otic capsule and SCC is identified and the superior otic capsule is drilled away until the blue line is visible at approximately 60° from the internal auditory canal (IAC). Focal micro-fenestration of the otic capsule is performed, and the aspiration needle is inserted through the fenestra. **(C)** Depiction of perilymph collection steps after sample aspiration into the needle to maximize efficiency and sample volume. Mass spectrometry can be used to assess the protein composition of perilymph, as previously described (Lysaght et al., 2011; Schmitt et al., 2021). I, incus; S, stapes. Medical illustrations are reproduced with permission from the Stanford Medicine Otologic Surgery Atlas (https://otosurgeryatlas.stanford.edu/) and the original artist Christine Gralapp, with text annotation added.

#### 3.5.3 Aspiration of perilymph with the needle

Expanding upon our previously reported method of retrieving perilymph from temporal bone plugs (Landegger et al., 2017), we depict the specific steps (Figure 4C) and provide a video demonstration (Supplementary Video 3). Due to the small total volume of perilymph, the aspirated sample is expelled directly from the needle into the collection tube for maximal yield. The needle is then detached from the syringe and placed in the same collection tube to maintain sterility of the needle tip. Next, 200 μL of 1x PBS is aspirated using the needleless syringe, the needle is reattached to the PBS-filled syringe, and the PBS expelled into the collection tube to flush the residual perilymph from the needle. The perilymph is then stored at −80 °C.

## 4 Discussion

This qualitative study describes practical refinements to postmortem human temporal bone procurement and perilymph collection intended to make this rare-specimen workflow more robust, reproducible, rapid, and transferable. The central technical insight is that the cause of circular saw blade fracture is multifactorial, reflecting interactions among blade design, the irregular MCF cutting surface, and operator-dependent alignment. Building on prior temporal bone plug procurement methods (Sagi et al., 2023), our direct observations, measurements, operator impressions, and retrospective accounts suggested that irregular MCF topography and non-orthogonal saw alignment may increase blade stress. These findings informed a modified blade design intended to allow staged engagement with cortical bone, guidance on saw-axis alignment and cortical scoring, and workflow refinements to reduce delays in perilymph collection.

These refinements should be interpreted in the context of existing temporal bone procurement approaches. En bloc lateral or subtotal skull base resection remains useful for medical education because it preserves native auricular anatomy and lateral surgical landmarks relied on intraoperatively during neurotologic procedures, uses standard autopsy equipment, and places conservative resection margins farther from the inner ear (Walvekar et al., 2010). For research autopsy workflows, however, this approach may cause greater cosmetic disruption, require longer procurement time, and yield larger specimens requiring prolonged processing such as decalcification. The circular temporal bone plug approach described by Nadol (1996) and revived by Sagi et al. (2023) is therefore better suited for rapid research procurement, but its broader use depends on reliable instrumentation, reproducible fabrication, operator training, and integration with time-sensitive workflows such as perilymph collection.

Our findings highlight the importance of treating procurement as an integrated workflow rather than a single osteotomy step. A blade that performs well on an idealized cutting surface may still fail when applied to the irregular MCF if the operator introduces off-axis loading or attempts to redirect the saw mid-osteotomy. Conversely, standardized cortical scoring and attention to body mechanics may reduce tool stress even when anatomic or table-height constraints cannot be changed. These teachable procedural elements are not typically emphasized in written temporal bone procurement protocols (Sagi et al., 2023; Nadol, 1996; Mass Eye and Ear, n.d.), which focus primarily on landmarks and saw placement, but they may be critical for training new prosectors and reducing preventable specimen loss. Proper body mechanics may serve as both fracture-prevention and efficiency measures because maintaining an orthogonal saw axis maximizes force transfer to the cutting surface, reducing wasted off-axis loading, and may shorten procurement duration. Consistent with this, experienced operators reported approximately 5 min per temporal bone after workflow optimization. Measured trainee procurement duration also progressively decreased across 5 serial sessions, consistent with improved workflow and operator experience. Given declining autopsy rates (Paratz et al., 2023; Harrington and Sayre, 2010), variable PMI, and the need for specialized expertise and locally fabricated tools (Sagi et al., 2023; Tucci and Doherty, 2019), the CAD-based blade design and visual training materials may facilitate standardization and adoption by other otopathology programs. The proposed refined saw design was based on qualitative observation and *in silico* compatibility; however, due to the unpredictable and limited number of research autopsies, fracture risk and performance of the refined blade have not yet been fully assessed. Thus, further technical validation is warranted to assess improved durability.

The perilymph workflow extends the value of temporal bone procurement by enabling collection of low-volume inner ear fluid before tissue fixation. Human perilymph is increasingly relevant for proteomic, inflammatory, and microRNA-based biomarker studies, but the opportunity to collect is time-sensitive and vulnerable to dilution, contamination, or logistical delay. Workflow refinements described here (e.g., early team communication, equipment readiness and redundancy, optimized specimen orientation, and efficient sample transfer) are simple but consequential because small delays can affect both the molecular sample and subsequent tissue preservation. Because perilymph sampling and fixation occur after plug harvest, faster procurement shortens the controllable component of the PMI for downstream histologic and molecular applications.

Certain limitations should be considered. Because this was a methods-development study using rare postmortem human specimens, the evidentiary basis varied by outcome. Preserving specimen integrity is the highest priority during procurement; therefore, limited quantitative data were feasibly collected. Quantitative data were limited to plug morphology and trainee procurement duration, whereas saw-handling impressions, historical timing, and prior fracture patterns relied on direct observation, operator reports, and retrospective accounts. These data informed practical workflow refinements but do not constitute controlled evidence of reduced fracture risk. Observations may also be subject to observer bias because operators and observers were aware of the study aims, and independent review was not possible per the video recording policy of the autopsy center. Retrospective accounts may be affected by recall bias, including incomplete recollection of fracture frequency, timing, and circumstances. Procurement-duration findings were descriptive and based on one operator across a few sessions. In addition, the low frequency of research autopsies has limited evaluation of the refined blade’s long-term durability and failure rate. Accordingly, the findings should be considered hypothesis-generating. Future studies should evaluate blade durability across larger numbers of procurements, assess operator learning curves before and after standardized visual training, and determine whether perilymph workflow refinements reduce time from harvest to sampling and improve downstream molecular data quality.

In conclusion, these refinements are intended to address practical barriers to postmortem human temporal bone procurement and perilymph collection. By integrating improved guidance on operator body mechanics, standardized cortical scoring, refined circular saw blade design, and optimized perilymph workflow, this approach is intended to reduce blade stress, improve procedural consistency, and maximize the scientific value of human inner ear specimens. These methods may help sustain and broaden access to human temporal bone otopathology for studies of hearing and balance disorders.

## Data Availability

Data supporting the findings of this study are available within the article and Supplemental Methods: Supplemental File 1, the Supplementary Videos are available on YouTube (Supplemental File 1), and the Supplementary CAD file is available on Zenodo (DOI: 10.5281/zenodo.20384380). Further inquiries can be directed to the corresponding author.
