# Supplementary material for: Refined circular autopsy saw design and standardized technique for postmortem human temporal bone procurement and perilymph collection
Source: Front Neurosci. 2026 Sep 18;20:1893053. doi: 10.3389/fnins.2026.1893053 (PMC13630995; doi:10.3389/fnins.2026.1893053)
Supplement: Supplementary file 1 [file Supplementary_file_1.docx]

**SUPPLEMENTAL VIDEOS AND URLS**

**Video 1. Overview of temporal bone procurement.** This video provides a brief overview of the anatomical landmarks, operator’s proper body mechanics, and one- and two-person protocol for postmortem temporal bone procurement. Link: https://youtu.be/wM45os2yFgk

**Video 2. Cochlear perilymph sampling procedure.** This video provides a surgical view of cochlear sampling via the transcanal approach. Link: https://youtu.be/wlBZBlDpn4o

**Video 3. Overview of the perilymph sampling process.** This video demonstrates the micro-volume transfer technique during the perilymph sampling, including use the aspiration needle and specimen collection tube for maximal volume retrieval. Link: https://youtu.be/H2lcJaMRck4.

**CAD file of the refined circular saw blade:** Zenodo DOI: 10.5281/zenodo.20384380.

**SUPPLEMENTAL METHODS**

**Representative workflow timeline for temporal bone procurement and perilymph collection**

A representative workflow timeline is provided to clarify the sequence of events during temporal bone procurement and perilymph collection. Patient death was followed by family consent for research autopsy and notification of the research team. The preparation interval depended on the expected postmortem interval (PMI) and autopsy classification: for rapid autopsies, the research team typically had approximately 3–10 hours to prepare before specimen procurement, whereas regular autopsies generally allowed >10 hours of preparation time.

At the start of the autopsy workflow, temporal bone procurement required ~10 minutes for setup, ~10 minutes for bilateral temporal bone osteotomy and soft-tissue resection, and ~10 minutes for cleanup. If perilymph was not collected, the temporal bone plug was placed directly into fixative after harvest. When perilymph collection was planned, the unfixed temporal bone plug was transferred on ice to the perilymph collection station, which required ~10 minutes of transport time. Bilateral perilymph collection required ~10 minutes, after which the temporal bone plug was placed into fixative and the perilymph sample was kept on ice. Perilymph samples were typically transferred to frozen storage ~10 minutes after sampling.

This timeline reflects the typical operational workflow rather than prospectively measured case-specific intervals for every donor. Because autopsy timing, staffing, specimen transport, and preparation requirements vary across cases, these intervals should be interpreted as representative estimates intended to clarify the sequence and approximate duration of each step.

**Additional methodological details for perilymph collection**

*Cochlear perilymph sampling via the transcanal approach*

For cochlear perilymph sampling, the transcanal round-window approach provides access to the scala tympani (**Figure 4A**). The temporal bone plug is mounted with the external auditory canal (EAC) positioned superiorly. The tympanomeatal flap is elevated using a round knife to expose the round window region and, if needed, the posterior medial EAC is enlarged using a 3 mm coarse diamond burr. The round window niche is drilled away using a 1 mm diamond burr to expose the entire round window membrane. After thorough saline irrigation to remove the residual bone dust, the perilymph aspiration needle is inserted through the round window membrane into the scala tympani just until the bevel is no longer visible, and perilymph is aspirated with the needle held stable. Surgical procedure of cochlear perilymph sampling is shown in **Supplemental Video 2**.

*Vestibular perilymph sampling via the superior semi-circular canal (SCC) approach*

For vestibular perilymph, the SCC approach provided access to vestibular perilymph from the middle cranial fossa (MCF) surface (**Figure 4B**). The temporal bone plug is oriented with the MCF surface superiorly and the dura is removed from the MCF floor using an osteotome. The arcuate eminence (AE) does not reliably overlie the SCC (31-36) and several intraoperative landmarks are absent from the temporal bone plug (22, 37, 38). Therefore, SCC location can be reliably estimated at approximately 60˚ from the internal auditory canal (IAC), even when the AE is displaced or absent. Copious saline irrigation and careful osseous reduction with a 3 mm diamond burr is performed with strokes parallel to but wider than the “blue line”, which is the translucent silhouette of the endosteum and membranous labyrinth of the SCC visible through the thinned otic capsule. Drilling continues with a 2 mm diamond burr, with periodic assessment of bone thickness by gentle palpation with a micro-pick. Once the bone becomes paper-thin, the MCF is copiously irrigated with saline and the thinned bone is carefully fenestrated with a micro-pick to expose the membranous labyrinth. The aspiration needle is inserted into the membranous labyrinth in the trajectory of the blue line, and the perilymph is aspirated.

*Materials*

Materials used for perilymph collection included osseous reduction surgical equipment (Medtronic Integrated Power Console, MN, USA), drill bits (Medtronic Midas Rex MR8, MN, USA), an aspiration-suction system, a 27G × 1.5-inch needle (BD PrecisionGlide Needle, catalog no. 301629, NJ, USA), a 1-mL tuberculin syringe (Covidien Monoject, catalog no. 1180100555, MN, USA), collection tubes (Eppendorf Safe-lock tubes, 1.5 mL, 0222993N, Hamburg, Germany), double-filtered 1× phosphate-buffered saline, pH 7.4 (Gibco, catalog no. 10010023, MA, USA), and ice.
